# Functional characterization of two novel 5' untranslated exons reveals a complex regulation of NOD2 protein expression

**DOI:** 10.1186/1471-2164-8-472

**Published:** 2007-12-20

**Authors:** Philip Rosenstiel, Klaus Huse, Andre Franke, Jochen Hampe, Kathrin Reichwald, Cornelia Platzer, Roland G Roberts, Christopher G Mathew, Matthias Platzer, Stefan Schreiber

**Affiliations:** 1 Institute of Clinical Molecular Biology, University Hospital Schleswig-Holstein, Campus Kiel, Germany; 2 Leibniz Institute for Ageing Research – Fritz-Lipmann Institute, Jena, Germany; 3 Institut für Laboratoriumsmedizin Berlin, Germany; 4 Department of Medical and Molecular Genetics, King's College London, United Kingdom

## Abstract

**Background:**

NOD2 is an innate immune receptor for the bacterial cell wall component muramyl-dipeptide. Mutations in the leucine-rich repeat region of NOD2, which lead to an impaired recognition of muramyl-dipeptide, have been associated with Crohn disease, a human chronic inflammatory bowel disease. Tissue specific constitutive and inducible expression patterns of NOD2 have been described that result from complex regulatory events for which the molecular mechanisms are not yet fully understood.

**Results:**

We have identified two novel exons of the *NOD2 *gene (designated exon 1a and 1b), which are spliced to the canonical exon 2 and constitute the 5' untranslated region of two alternative transcript isoforms (i.e. exon 1a/1b/2 and exon 1a/2). The two novel transcripts are abundantly expressed and seem to comprise the majority of NOD2 transcripts under physiological conditions. We confirm the expression of the previously known canonical first exon (designated exon 1c) of the gene in unstimulated mononuclear cells. The inclusion of the second alternative exon 1b, which harbours three short upstream open reading frames (uORFs), is downregulated upon stimulation with TNF-α or under pro-inflammatory conditions in the inflamed intestinal mucosa *in vivo*. Using the different 5' UTR splice forms fused to a firefly luciferase (LUC) reporter we demonstrate a rapamycin-sensitive inhibitory effect of the uORFs on translation efficacy.

**Conclusion:**

The differential usage of two alternative promoters in the *NOD2 *gene leads to tissue-specific and context-dependent *NOD2 *transcript isoform patterns. We demonstrate for the first time that context-dependent alternative splicing is linked to uORF-mediated translational repression. The results suggest complex parallel control mechanisms that independently regulate NOD2 expression in the context of inflammatory signaling.

## Background

NOD2 (CARD15, NLRC2) is a member of the family of the NACHT/LRR receptors, which are characterized by a central nucleotide-binding and oligomerization domain and a C-terminal sensor domain consisting of repeats of leucine-rich repeats (LRR) [1,2]. The LRRs of NOD2 have been described to directly or indirectly recognize intracellular muramyl-dipeptide (MDP), an abundant cell wall component of both gram-negative and gram-positive bacteria [3,4]. The recognition of MDP leads to a recruitment of the protein kinase RIP2/RICK to the N-terminal effector binding domain, which consists of two adjacent caspase-recruitment domains (CARDs). Subsequently, the canonical IKK/IκB/NF-κB pathway is activated via induced proximity signaling [5]. NOD2 has been identified as the first major susceptibility gene for Crohn's disease (CD) [6-8]. Three genomic variations within the *NOD2 *gene, one frameshift (*rs2066847*, L1007fsinsC) and two missense mutations (*rs2066845 *– G908R, *rs2066844 *– R702W) represent the main causative functional variants and are associated with a deficient activation of the transcription factor nuclear factor-kappa B (NF-κB) upon microbial triggering [6-10]. It has recently been shown that pro-inflammatory stimuli, such as TNF-α, IFN-γ and lipopolysaccharide (LPS) activate NOD2 (CARD15) gene expression in intestinal epithelial cell lines and primary intestinal epithelial cells as well as monocytic HL-60 cells [11,12]. This up-regulation is at least in part dependent on the binding of NF-κB to a proximal κB-binding element (-26) of a *NOD2 *promoter region in front of the canonical first exon.

While performing the mutation detection and gene model verification of *NOD2/CARD15*, we performed cross-species comparisons and EST database analyses that indicated an incomplete annotation of the 5' region of the gene. We have thus investigated the genomic region of the NOD2 (CARD15) locus for the evidence of additional upstream exons. We show that two additional untranslated exons exist located upstream of previously described first exon of the NOD2 (CARD15) gene, which are present in transcript isoforms that exclude the previously reported canonical exon 1. These two exons exhibit a distinct splicing pattern under pro-inflammatory conditions both *in vitro *(monocytic cells) and in colonic biopsies from patients with chronic inflammatory bowel disease (IBD) *in vivo*. A long isoform, which contains both novel exons, contains three short upstream open reading frames (uORFs), which are shown to inhibit translational efficacy of the respective transcript. The results provide evidence for a complex regulation of NOD2 expression that includes the alternative usage of two promoters and post-transcriptional regulation of translation by uORFs.

## Results

### Database and inter-species comparisons of the NLR family

Database sequences and cross-species comparisons indicated that the current annotation of the 5' – part of the *NOD2/CARD15 *transcript is incomplete: The *CARD15 *gene structure as annotated in the Genbank record AF178930 was comprised of 12 exons with the translational start in exon 1 and the stop codon in exon 12. The Northern analysis as reported by Ogura showed two transcripts in peripheral blood leukocytes [5]. Furthermore, CARD15 mRNA isoforms in the databases differ in their 5' part and in the number of exons (AF178930[5]; AJ303140 [6]).

A comparison of the 5'UTRs of the CARD15 mRNA species and those of the other members of the NOD family, APAF-1 and CARD4 (NOD1) indicated striking differences. Both APAF-1 (577 nt) and CARD4 (424 nt) transcripts exhibit much longer 5'UTRs as compared to 105 or 146 nucleotides in the deposited CARD15 mRNAs. Inspection of the genomic locus of CARD15 revealed another discrepancy in the locus organization of CARD15. While the first exons of APAF-1 and CARD4 are located in CpG islands and their positions are predicted at the respective genomic loci by a commonly used promoter/first exon prediction program, the 5' end of the CARD15 gene is not found in such a context. The gene structures of CARD4 and CARD15 indicate that the two genes are paralogues, because their exon/intron architecture is very similar with respect to both exon sizes as well as intron phases. This similarity is not present in the 5' parts of the genes.

An interspecies comparison of the human (NM_022162.1) and murine (NM_145857.2) and bovine (NM_001002889.1) transcripts of *CARD15 *shows a lack of homology upstream of exon 2 (Suppl. Fig 1).

### Additional upstream exons of the CARD15 gene

**Figure 1:**
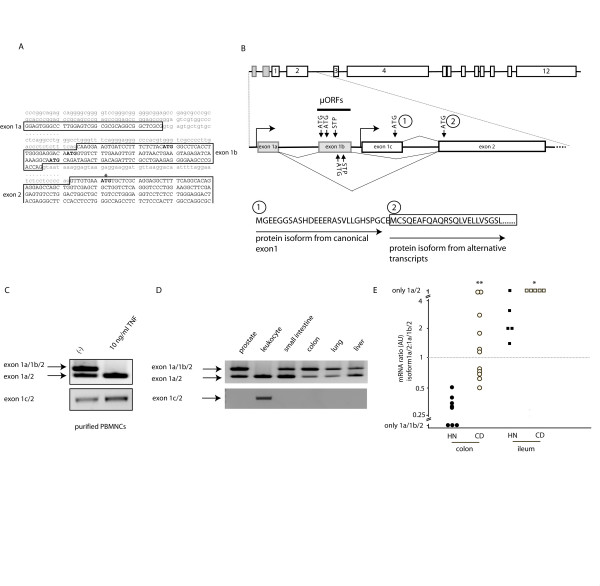
**Structure of the 5'region of the human NOD2 gene and expression of transcript isoforms**. (A) Sequence of the novel two exons in the genomic context. Exons are capitalized, ATGs representing upstream ORFs are in bold print. The productive ATG used as an alternative translation start in exon 2 is marked by an asterisk. (B) Graphical representation of the alternatively spliced isoforms from the two alternate promoters. STP, stop codon of the uORFs. Below is a representation of the alternatively translated protein isoforms depicted by single letter amino acid code. (1) denotes the original start in the canonical exon 1, (2) denotes the alternative start in exon 2 used by the two novel transcript isoforms. (C) Primary monocytes were treated with TNF-α (10 ng/ml) for 12 h. NOD2 was amplified as outlined and analyzed on agarose gels (D) Amplification of NOD2 in a human multiple tissue panel. Note the preponderance of the short isoform exon1a/2 in the leukocytes. (E) Densitometric analysis of RT-PCR experiments from colonic biopsies of healthy controls and inflamed tissue from Crohn disease patients. Depicted is the ratio between the short (exon1a/2) and the long (exon 1a/1b/2) isoform of NOD2 (**p < 0.002; *p < 0.05, Student's T-test, colonic samples from *n *= 8 healthy controls and *n *= 11 inflamed Crohn disease patients;*n *= 5 ileal samples from healthy controls and *n *= 5 inflamed ileal samples from Crohn disease patients).

We scanned 13 kb upstream of exon 2 with the FirstEF program [13]. The program detected no CpG island in front of the previously described exon 1. The prediction program detected a promoter and first exon within a CpG island about 3.5 kb upstream of exon 1 of the CARD15 locus. RT-PCR with primers located in this predicted exon and in the known exon 2 was performed. Two amplicons were obtained from cDNA of peripheral blood leukocytes (Figure 1A). Cloning and sequencing revealed that the smaller species corresponds to the predicted first exon spliced to exon 2 of CARD15. The larger amplicon contained an additional exon included between the new exon 1 and exon 2, which corresponded to an exon predicted to belong to an independent *grailed*-homology transcript [6]. Canonical splice sites are flanking the intervening sequences on the genomic DNA (Figure 1B). We name these newly identified exons 1a and 1b and keep the designation (exon1) for the formerly described first exon (as in AF178930). These exons were also confirmed by 5'rapid amplification of cDNA ends (RACE) using a gene specific reverse primer in exon 2 and sequences were deposited in the NCBI database (AY187245, short alternative 5'UTR; AY187242, long alternative 5'UTR; AY187243, canonical exon1). The short alternative 5'UTR has also been found in a systematic approach characterizing transcripts, which contain alternative promoter regions (DA224866 in [14]).

RT-PCR using sense as well as antisense primers located in exon 1 in combinations with the respective forward and reverse primers in exon 1a and 1b did not detect an amplicon containing exon 1c together with the novel exons in cDNA derived from human monocytes and in cDNAs from various organs (heart, brain, placenta, lung, liver, skeletal muscle, kidney, pancreas, spleen, thymus, prostate, testis, ovary, small intestine and colon) (*data not shown*). PCR amplification with primers located in exon 1c gave a positive result when using reverse primers in exon 2, 3 or 4. The results taken together with other findings that confirm the existence and/or induction of the canonical exon 1 [5,11,15] point to the usage of two alternative promoters.

### Distribution and regulation of splice isoforms

To identify expression patterns of the alternative transcript isoforms, we analyzed expression in adult human tissues by RT-PCR methods using primers spanning exon1a to 4. The identity of the resulting amplicons was confirmed by DNA sequencing (not shown). The RT-PCR analysis showed that the mRNA for the alternative NOD2 isoforms are abundantly expressed in the small intestine, colon, prostate, lung and in liver. Interestingly, the ratio between the long and the short isoform varied considerably, e.g. peripheral blood leukocytes showed a striking preponderance of the short exon 1a/2 isoform (alt 5'UTR NOD2 a, Fig. 1D).

To analyze whether the ratio of the different UTR mRNA isoforms of NOD2 is regulated by cytokine stimulation, primary human monocytes were purified and stimulated for 12 h with TNF-α (10 ng/ml). Transcripts encoding novel isoforms of NOD2 were amplified as outlined and analyzed on an agarose gel (Fig. 1C). Here, the stimulation of resting monocytes led to a decrease of the long (exon1a/1b/2; alt 5'UTR NOD2 b) isoform to nearly undetectable levels, whereas the levels of transcripts encoding the alternative short form were increased. The transcript ratio of the UTR isoforms of NOD2 upon stimulation was further analyzed by cloning and counting of PCR products (> 100 individual clones) as previously described [16,17]. The percentage of clones containing the short form of the UTR was similarly increased upon TNF-α stimulation (*data not shown*).

A pronounced increase of the short UTR isoform was detectable in inflamed colonic and ileal biopsy specimen from patients with Crohn disease. Using a densitometric analysis of agarose gels of colonic samples, the short form (exon1a/2) was significantly overrepresented in inflamed CD biopsies, whereas in control subjects, which underwent colonoscopy for routine cancer surveillance, four to five times more of the long isoform (exon 1a/1b/2) was detected (Fig 1E, p < 0.002, Student's T-test). In contrast, in non-inflamed biopsies from healthy controls higher levels of the short form (exon1a/2) were observed than in the non-inflamed colonic samples. However, in inflamed ileal biopsies of Crohn disease patients only the short form (exon 1a/2) was detectable (Fig. 1E, p < 0.05).

### Upstream ATGs in exon 1b inhibit translation efficiency

**Figure 2:**
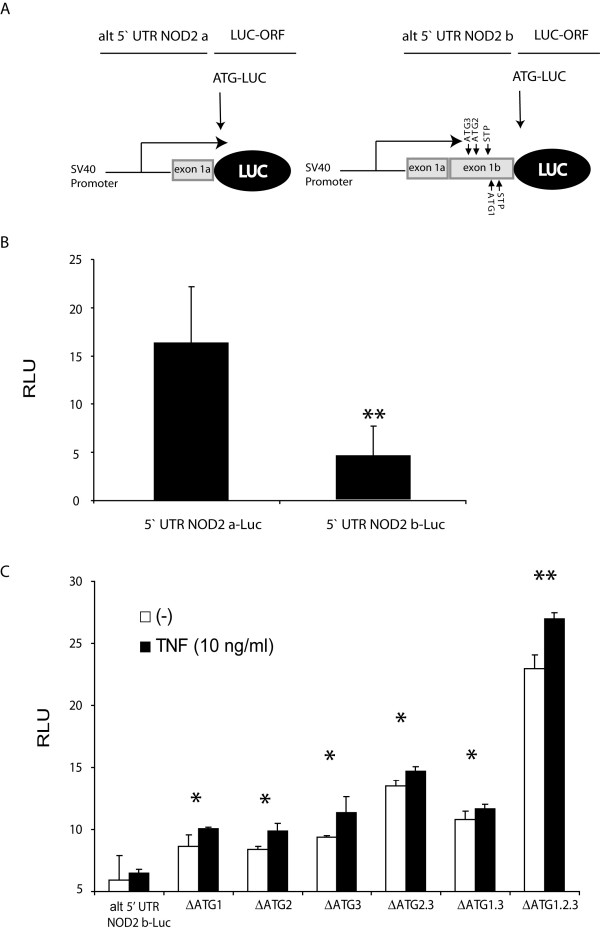
**Upstream ORFs (uORFs) in the 5'UTR of the alternative NOD2 transcripts have an effect on translational efficacy**. (A) Reporter gene constructs containing the differentially spliced alternative 5'UTRs of NOD2 (alt 5' UTR NOD2 a, containing only exon 1a and alt 5' UTR NOD2 b containing exon 1a and 1b) cloned in front of a luciferase reporter gene (pGL Basic) ATG1-3 denote the uAUGs and STP the respective stop codons. (B) Constitutive luciferase activities of the two constructs in transfected HEK293 cells. (C) Sequential deletion of the uAUGs (e.g. ΔATG 1 denotes deletion of the first uAUG) abolishes the inhibitory activity of the long alternative 5'UTR (alt 5' UTR NOD2 b). All values correspond to mean ± SD calculated from at least three independent experiments. RLU, relative luciferase units, determined by dual luciferase assay *p < 0.05, **p < 0.01, Student's T-Test).

**Figure 3:**
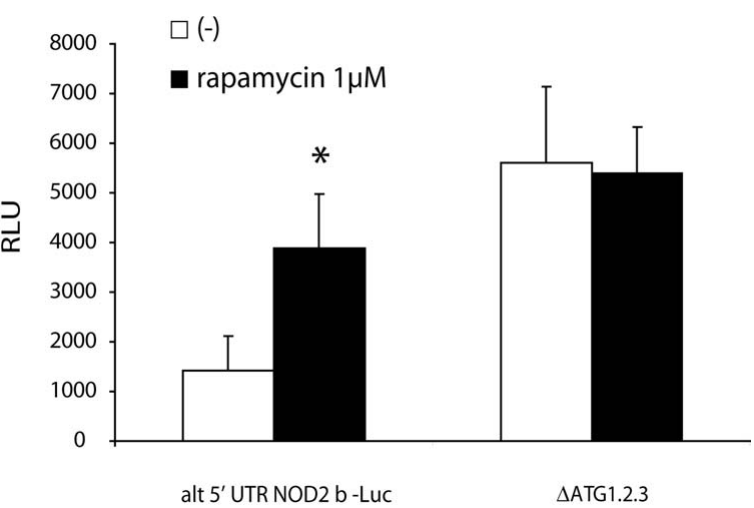
**Translational inhibition of the alternative NOD2 5'UTR can be inhibited by rapamycin**. Relative luciferase activities were determined from the construct containing both 5'UTR exons with and without deletion of the uAUGs (ΔATG1.2.3 and wildtype). Note the significant reduction of the inhibitory effect of the uORFs after rapamycin treatment (1 μM). Values represent mean ± SD calculated from three independent experiments. RLU, relative luciferase units, determined by dual luciferase assay *p < 0.05, Student's T-Test).

Sequence inspection suggested that the NOD2 mRNA isoform with exon1a being joined directly to exon 2 (*alt 5'UTR NOD2 a*) allows translation from the very first ATG localized in exon 2. The longer isoform (Exon1a/1b/2, *alt 5'UTR NOD2 b*) appeared to exhibit structural features which may interfere with efficient translation. Exon 1b contains three additional upstream ATGs (uATGs), the first two being in the same phase with a stop codon and the subsequent CARD15 start, while the third one begins a new upstream ORF (uORF) in a different reading frame. No significant similarity to other known proteins was found in the 14 amino acid peptide (MGLTLGRTMVSLKL) resulting from the first uORF in the non-redundant (nr) sequence database using blastp and blastx algorithms. The second uORF encodes a six amino acid (MVSLKL) peptide, the third start methionie results in an uORF of 4 amino acids (MQID). uATGs and short uORFs are considered as regulatory elements for expression control on the level of translation. We inserted the UTRs upstream of the exon 2 ATG of the two newly identified splice forms into a reporter gene vector in order to monitor differential translation efficiency by dual luciferase assays (Figure 2A). HEK 293 cells and HL-60 monocytic cells (*not shown*) transfected with the short form (*alt 5'UTR NOD2 a*) exhibit up to 4-fold higher luciferase activities than cells transfected with the vector containing the longer isoform (*alt 5'UTR NOD2 b*; Figure 2B). Using sequential deletion constructs of the three uATGs (ΔATG 1–3) generated by site-directed mutagenesis we could demonstrate that the translation effiency from the same construct was significantly increased when the uORFs were removed (Figure 2C). Similarly, treatment of *alt 5'UTR NOD2 b-Luc *transfected cells with the mTOR inhibitor rapamycin led to an increase of luciferase activity (Figure 3) suggesting a role for an mTOR mediated modulation of the translation initiation factors eIF2 α and eIF4E in the control of the uORF-mediated translational repression of NOD2 as reported for other uORF-containing UTRs [18-22].

## Discussion and Conclusion

The salient finding of the present study is the demonstration of two novel exons (exon1a and 1b) in the *NOD2/CARD15 *gene, which appear to contribute to the tight regulation of NOD2 protein levels in the physiological setting. Expression of transcripts containing exon1a and 1b is driven by an alternative promoter located within a CpG island, whereas transcripts containing the canonical exon 1 are regulated by an NF-κB-dependent promoter. Evidence is increasing that the 5'-UTR of eukaryotic mRNA pivotally contributes to post-transcriptional regulation of gene expression [23-26]. AUG codons (uAUG) upstream of the main open reading frame or short upstream ORFs are present in approx. 10% of all mRNAs [27]. Although the functional impact of this mechanism has not been investigated on a genome-wide level, recent studies on selected UTRs containing uAUGs or uORFs suggest an important role in negative translational control across kingdoms from plants to mammals [28-32]. Within the exon 1b of the alternative 5'-UTR region of human *NOD2*, three uORFs with variable lengths were identified upstream of the main ORF located in exon 2. These uORFs exert a negative influence on translational efficacy as shown by luciferase reporter gene assays and site-directed mutagenesis of the uAUGs. The effect was rapamycin-sensitive pointing to a role of the eukaryotic translation initiation factors eIF2α and eIF4E, which were shown to be decisive factors for regulated initiation of translation of a subset of mRNAs that have *cis*-regulatory small upstream open reading frames [18,22,33]. Although a tendency towards higher luciferase expression was observed for the reporter construct representing the short novel isoform after stimulation with the proinflammatory cytokine TNF-α, further investigations are warranted to identify signaling pathways involved in adjusting the activity of elFs in acute and chronic inflammation.

We demonstrate for the first time that context-dependent alternative splicing is linked to uORF-mediated translational repression. This additional regulatory mechanism may to contribute to the overall control of NOD2 protein level in inflammation. We show that stimulation of monocytic cells with the pro-inflammatory cytokine TNF-α shifts the ratio of the splice isoforms towards the shorter isoform that lacks exon1b containing the regulatory uORFs. Similarly, in the inflamed colonic tissue of Crohn disease patients, a preponderance of the short alternative 5'UTR transcript isoform could be detected, which may result in higher protein levels in the absence of uORFs. The three main variants in *NOD2 *(*rs2066847 *– L1007fsinsC, *rs2066845 *– G908R and *rs2066844 *– R702W) are genetically associated with a distinct subphenotype of CD involving the small intestine (Vienna Classification L1 and L3 [34]) [35,36]. High constitutive expression of NOD2 has been shown in Paneth cells [37], which represent specialized IECs secreting antimicrobial peptides in the terminal ileum. A genetic ablation of *Nod2 *in mice leads to a distinct defect in cryptdin (α-defensin orthologues in mice) expression in the ileum [38]. Consistently, a NOD2-genotype dependent impairment of human α-defensin -5 and -6 has been shown in ileal CD[39], which may pivotally contribute to the immunological barrier dysfunction observed in CD etiopathogenesis [40]. Interestingly, in biopsies from the ileum we observed a higher constitutive representatation of the short (i.e. translationally more active exon 1a/2) isoform. In the inflamed ileum only the exon1a/2 form was detectable. We thus hypothesize that alternative splicing of the two novel isoforms contributes to the upregulation of NOD2 protein levels observed under inflammatory conditions and the high constitutive expression in distinct cell types, i.e. monocytic cells and, possibly, Paneth cells. Interestingly, stimulation of monocytes with TNF-α results in a downregulation of a short alternatively spliced isoform of NOD2 encoding an auto-inhibitory CARD-only protein, which is generated by skipping of exon 3 [17]. Albeit reciprocal mechanisms involved (increased vs. decreased exon skipping), these complex changes in splice patterns of NOD2 transcripts after TNF-α stimulation all result in a higher sensitivity of the cellular NOD2 sensor function.

An obvious paradoxon is created by the fact that "loss-of function" variants in *NOD2 *have been genetically associated with a higher risk for the development of Crohn disease, whereas higher levels of NOD2 protein have been detected by several groups independent from the respective *NOD2 *genotype. However, it is generally accepted that up-regulation of NOD2 expression in intestinal inflammation is a generic response to bacterial invasion and represents a futile attempt to restore the disrupted epithelial barrier in patients with Crohn disease. It will be a major goal to dissect the contribution of the differential usage of the two alternative promoters in different cell types and the regulation of the uORF-mediated translational repression to the overall control of NOD2 protein level. These studies may not only provide deeper insights in the pathophysiology of human inflammatory disorders, but may also help to understand the complex physiological regulation of sensitivity and tolerance of the innate immune system during the life-long interaction with the commensal flora.

## Methods

### Exploration of the 5' region: computer analyses, RT-PCR

The genomic sequence of the CARD15 locus (Genbank: AF178930) was analyzed using computer prediction methods as described in [13]. In order to verify the published *NOD2 *gene model, conserved exons were searched for in mouse, cow and human genomic sequences (Twinscan gene predictions [41]) and related to predicted splice sites [42]. 5'exons of the different species were aligned using ClustalW [43]. The previously reported and the predicted exons were verified by RT-PCR and by subsequent cloning and sequencing of PCR products: (i) in a pool of cDNA tissue samples, (ii) in cDNA derived from freshly isolated monocytes and (iii) in cDNA from colonic biopsies.

### Monocyte isolation & colonic biopsy samples

Monocytes were isolated from 100 ml of peripheral blood drawn from four healthy volunteers (age range 24 – 33; 1 female) and cultured as described previously [44]. CD Patients (*n *= 11 for colonic biopsies, n = 5 for ileal biopsies) and normal controls (*n *= 8 for colonic biopsies and n = 5 for ileal biopsies) were recruited at the Department of General Internal Medicine in Kiel, Germany. Up to six additional biopsies were obtained from inflamed regions as part of a routine colonoscopy indicated on clinical grounds. The diagnosis of Crohn's disease was assigned on the basis of established clinical, endoscopic and radiological findings. Normal control biopsies were obtained from patients, who had a macroscopically and histologically normal colon, during procedures performed for the investigation of unspecific abdominal pain or cancer screening. All individuals gave written, informed consent for extra biopsies and/or anonymized genetic and functional analysis at least one day before the procedure. The study and sample collection was approved by the institutional ethics board before initiation of the study.

Biopsies were snap frozen in liquid nitrogen immediately after sampling. Snap-frozen biopsies were crushed to a fine powder under liquid nitrogen using a manual crusher with a teflon head (Omnilab, Bremen/Germany) and total RNA was isolated using the RNeasy Mini Kit (Qiagen, Hilden/Germany) according to the manufacturers protocol. Eluted RNA was determined to be intact as assessed by standard formamide gel electrophoresis and shown to be free of contaminating genomic DNA when primers specific for GAPDH yielded a PCR product only after reverse transcription.

### mRNA isolation and RT-PCR

Total RNA from primary peripheral blood monocytes was isolated using the RNeasy kit from Qiagen. 300 ng of total RNA were reverse transcribed as described elsewhere [44]. For investigation of tissue specific expression patterns, a commercial tissue panel was obtained from Clontech (Palo Alto, CA, USA). Primers used for amplification of the alternative NOD2 transcript were (5'-CAC TGG GCT TTT GGC GTT C-3' (sense) and 5'-CGG CAA CCT GAT TTC ATC AC-3' (antisense). Primers used for amplifying the canonical transcript were 5'-GGA GTG GGC CTT GGA GTC GG-3' (sense) and 5'-CCA GGA CAT TCT CTG TGT ATA T-3' (antisense).

The following conditions were applied: Denaturation for 5 min at 95°C; 35 cycles of 30 sec at 95°C, 20 sec at 60°C, 45 sec at 72°C; final extension for 10 min at 72°C. To confirm the use of equal amounts of RNA in each experiment all samples were checked in parallel for β-actin mRNA expression. All amplified DNA fragments were analyzed on 1% agarose gels and subsequently documented by a BioDoc Analyzer (Biometra, Göttingen, Germany).

### Quantification of splice variants

To determine the exact ratio of transcripts containing exon1a/1b/2 vs. exon 1a/2, we employed a clone sequencing method that has been described to unveil genotype-splicing effects [11,16,45]. PCR products from 30 rounds of PCR (exponential phase) were ligated into a TA-cloning vector (Invitrogen) and the indicated numbers of individual clones were sequenced using Dye terminator chemistry (Applied Biosystems, Foster City, CA, USA) on a 3730 × L DNA Analyzer (Applied Biosystems).

### Reporter assays

The 5'ÚTR of the alternative NOD2 transcript was amplified from cDNA by PCR under standard conditions with the following primers (restriction sites underlined) NOD2HindIIIpGL: AAGCTTGGAGTGGGCCTTGGAGTC und NOD2NcoIpGL: CCATGGACATTTCACAACCTGGTCG and cloned into a TA vector (Invitrogen). After HindIII/NcoI restriction the inserts were subcloned into a pGL2-luciferase plasmid (Promega). All constructs were sequence-verified in an ABI3730xL sequencer prior to use. Modifications of the UTR were performed with the site-directed mutagenesis kit from Stratagene according to the manufacturer's instructions. All primers were purchased from Eurogentec (Liège, Belgium).

HL-60 myelomonocytic and HEK293 cells were purchased from the German Collection of Microorganisms and Cell Cultures (DSMZ, Braunschweig, Germany). All cells were cultured in RPMI + 10% fetal calf serum (FCS). Transfections were performed with Fugene 6™ (Roche, Basel, Switzerland) using indicated amounts of the respective plasmids. Medium was changed 4 h after the transfection to avoid transfection reagent presence at the time of stimulation. TNF-α was purchased from R&D Systems (MN, USA), rapamycin was from Sigma (Deisenhofen, Germany). Normalized luciferase activity was determined with a Dual Luciferase Reporter gene kit (DLR) from Promega using the the *Renilla*-containing plasmid pRL-TK driven by the thymidin kinase minimal promoter. Cell lysates were analyzed with a Tecan Genios Pro (Tecan, Bubendorf, Switzerland). All samples were at least measured in duplicates and are results of three independent experiments. The results for firefly luciferase activity were normalized to renilla luciferase activity.

## Abbreviations

CARD; caspase recruitment domain

IκB; nuclear factor of kappa light chain gene enhancer in B-cells inhibitor

MDP; muramyl dipeptide

NF-κB; transcription factor nuclear factor kappa B

NOD; nucleotide-binding and oligomerization domain

RIP2; receptor interacting protein kinase 2

TNF-α, tumour necrosis factor alpha

uORF, upstream open reading frame

uAUG, upstream start codon

## Authors' contributions

PR, KH, MP and SS designed the study. PR, KH, KR, CP performed the laboratory work. PR, AF, KH, JH, MP and SS performed data analysis. Interpretation of data and writing of the manuscript were done by PR, KH, RR, CM, MP and SS. All authors read and approved the manuscript.
